# Molecular Mechanisms of Plant Stress Memory: Roles of Non-Coding RNAs and Alternative Splicing

**DOI:** 10.3390/plants14132021

**Published:** 2025-07-02

**Authors:** Mariz Sintaha

**Affiliations:** Department of Life Sciences, Independent University, Bangladesh, Dhaka 1229, Bangladesh; sintu.bmb@gmail.com

**Keywords:** stress memory, transgenerational stress, stress priming, miRNA, siRNA, lncRNA, alternative splicing, RdDM

## Abstract

The ability of plants to protect themselves from stress-related damages is termed “adaptability” and the phenomenon of showing better performance in subsequent stress is termed “stress memory”. This phenomenon has been reported in various stresses such as drought, heat, salinity, cold, and heavy metal toxicity. Histone modification leading to chromatin remodeling and accumulation of phosphorylated RNA polymerase on the promoters of memory genes is a well-known mechanism of plant stress memory. Recent studies have revealed the role of non-coding RNAs (ncRNAs) and alternative splicing (AS) in memory-specific gene expression and transgenerational inheritance of stress memory. MicroRNAs (miRNAs) inhibit specific genes to enable plants to respond better in subsequent drought and heat stress, while long non-coding RNAs (lncRNAs) play roles in epigenetic regulation of memory gene expression in cold and salt stress. Small interfering RNAs (siRNAs) lead to DNA methylation during the memory response of biotic, salt, and heavy metal stress. Simultaneously, stress-responsive isoforms of tolerant genes are found to be expressed as a memory response in subsequent heat stress. This review highlights the stress-type-specific roles of ncRNAs and AS in establishing, maintaining, and transmitting stress memory, offering insights into their potential for improving crop resilience through genetic and epigenetic priming strategies.

## 1. Introduction

Plants are likely to endure various stresses during their lifetime. The ability of plants to respond effectively to environmental stress is termed “adaptability” and the phenomenon of showing better performance in terms of adaptability in subsequent stresses, compared to the plant experiencing the first stress, is termed “Stress Memory”. Plants’ memory response can be observed in terms of physiological response, biochemical response, gene expression, or epigenetic changes. The term “memory” implies not only the repeated observation of a response but a modified magnitude or speed of the response, indicating a primed physiological state. The existence of memory also allows plants to respond more rapidly and effectively to subsequent stress events.

Stress memory has been reported for various stresses including drought [1], heat [2], salinity [3], cold [4,5], and heavy metal [6,7] stress, among others. *Arabidopsis thaliana* (L.) Heynh. plants primed with dehydration and abscisic acid (ABA) treatment show stress memory [8]. The stomata remain partially closed in subsequent stresses to reduce water loss through transpiration, resulting in high relative water content in leaves [8,9]. *Aptenia cordifolia* (L.f.) Schwantes has been found to change its photosystem structure in preparation for future stress and maintain the chlorophyll a/b ratio during repeated drought stress [10]. A similar phenomenon was observed in *Silene dioica* (L.) Clairv. [10]. In wheat and potato, drought priming before the flowering stage resulted in higher grain yield and tuber yield, respectively [11,12]. In rice and maize, drought priming was found to reduce stomatal conductance and photosynthesis during subsequent stress and in rice, and it was restored rapidly during rewatering [13]. In wheat, potato, rice, and maize, drought memory response differs between different genotypes, indicating that the memory response is elicited in a genotype-specific manner [11,12,14]. In drought-sensitive olive plant and wheat plants, priming results in higher activity of superoxide dismutase (SOD), catalase (CAT), and glutathione peroxidase (GP) enzymes to maintain reactive oxygen species (ROS) homeostasis [11,15]. In some plants, such as *Cakile maritima* Scop. and Arabidopsis, a cross-tolerance response has been observed where priming with one type of stress results in tolerance to multiple types of stress [16,17].

Transgenerational stress memory in plants refers to the phenomenon in which plants transmit the effects of stress experienced by their ancestors to subsequent generations. For example, heavy metal stress memory is found in rice where the methylation pattern of a heavy metal-responsive gene is altered and transported to the next generation with increased expression compared to the first generation [6]. In *Arabidopsis*, the progenies of heavy metal-primed parents show higher tolerance not only to heavy metal stress but also to salt and methyl methane sulfonate (MMS) stress, demonstrating a transgenerational and cross-tolerance memory in this genus [18]. In *Arabidopsis*, temperature and UV-B stress induced alteration in histone acetylation but it was heritable only in a small number of cells [19]. Other plants that showed transgenerational memory include dandelion [20], tobacco [21], wheat [22], and tomato [23].

Stress memory response is elicited in plants by the changes in the expression of genes between the two cycles of stress. A genome-wide RNA-sequencing approach identified that the memory response can be reflected in both increasing and decreasing patterns of gene expression [24,25]. The underlying mechanism of change in gene expression includes modification of histone molecules associated with these genes. In *Arabidopsis*, the higher expression of dehydration-responsive genes (*RD29B*, *RAB18*) in subsequent drought stress was found to be associated with higher histone 3 lysine 4 trimethylations (H3K4me3) [26]. Maize also showed transcriptional memory for the homologous genes of *Arabidopsis*’ memory genes as well as associated histone modification [27]. In soybean, salt-primed seedlings were found to have altered histone 3 lysine 4 demethylation (H3K4me2), histone 3 lysine 4 trimethylation (H3K4me3), and histone 3 lysine 9 acetylation (H3K9ac) marks throughout the genome to promote the response related to salt tolerance [28]. The phosphorylation pattern of RNA polymerase II also affects gene expression during repeated stress. The serine residue present in the consensus (YSPTSPS) repeats in the C-terminal domain of RNA polymerase II must be phosphorylated (Ser5p Pol II) for the elongation step of transcription to be carried out, which is removed after the transcription is complete [29]. In *Arabidopsis*, serine phosphorylation of RNA polymerase II is not removed after the stress subsides, and thus, Ser5p Pol II is stalled during each recovery stage, ensuring the rapid transcription of the memory gene in subsequent stress [26].

Recent studies have found that, besides histone modification and RNA polymerase II phosphorylation, alternative splicing mechanisms and various non-coding RNAs such as miRNA, lncRNA, and siRNA also play roles in plants’ stress memory. This article aims to summarize the role of non-coding RNAs in drought, cold, heat, heavy metal, salt, and biotic stress memory.

## 2. Non-Coding RNAs and Alternative Splicing

In many plant species, particularly those with large genomes such as maize or wheat, a major portion—often exceeding 80%—of the genome consists of intergenic regions, repetitive sequences, transposable elements (TEs), and pseudogenes. The non-coding RNAs are products of these regions and genic regions which are not translated into proteins and are known as non-coding RNAs (ncRNAs). These ncRNAs can be classified as siRNA (small interfering RNA), microRNA (miRNA), and long non-coding RNA (lncRNA). In plants, major types of ncRNAs are miRNAs and siRNAs [30], which are produced from plant TEs containing stress-responsive cis-acting elements in promoter regions [31]. These ncRNAs regulate gene expression through diverse mechanisms, including mRNA degradation, translational repression, chromatin modification, and scaffolding of protein complexes [32].

Plant siRNAs are usually 20–25 nucleotides long [33]. They are transcribed as double-stranded RNA precursors but later converted to smaller siRNA duplexes by DICER-LIKE 3 (DCL3) protein [34,35], one strand of which becomes associated with a protein complex known as RNA-induced silencing complex (RISC). RISC contains AGO protein, which acts as the catalytic component [36]. This complex finds and binds mRNA with a complementary sequence so that AGO protein can cleave it. Therefore, binding of siRNA to its target mRNA leads toward the destruction of mRNA, which causes the post-transcriptional silencing of gene expression [37].

**Figure 4:**
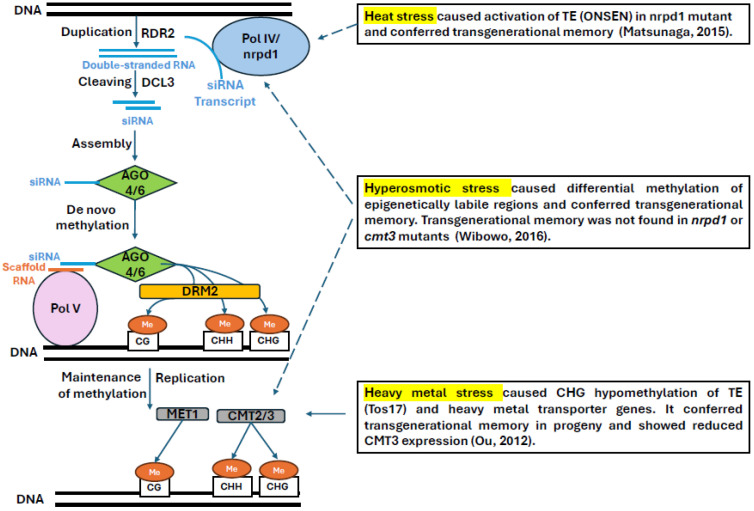
Role of RdDM in transgenerational epigenetic inheritance of stress memory [88,92,93].

Alternatively, siRNA can cause cytosine methylation at loci containing their homolog sequences, thereby causing suppression of transcription. This process is known as RNA-directed DNA methylation (RdDM) [38]. RdDM plays a role in transcriptional gene silencing, transposable element suppression, genome stability, developmental regulation, responses to abiotic and biotic stresses, and transgenerational epigenetic inheritance [39]. In RdDM, a siRNA transcript is produced by Nuclear RNA Polymerase D (NRPD or RNA polymerase IV), which is converted into double-stranded RNA by RDR2 and then processed into 24 nt siRNAs by DCL3. These siRNAs become associated with their effector protein AGO4 or AGO6. AGO4/6-siRNA complex enters the nucleus so that siRNA of this complex can base pair with its homologous transcript (scaffold RNA) produced by RNA polymerase V. This interaction helps guide the complex to specific genomic regions. The siRNA–scaffold RNA pairing facilitates the recruitment of DOMAINS REARRANGED METHYLTRANSFERASE 2 (DRM2) at these RdDM loci which causes de novo cytosine methylation (CG, CHG, and CHH where H is A, C, or T) [38,40,41,42]. After DNA replication, CG methylation is maintained by MET1 and CHG/CHH by CMT3 and CMT2. The maintenance of CHG methylation is closely linked to histone H3K9 di-methylation, which is added by the histone methyltransferase KYP (KRYPTONITE). This mutual reinforcement between DNA and histone methylation helps to stably silence gene expression [43]. CG and H3K9 methylation together downregulate gene expression. The ability of siRNA to move between cells through the plant vasculature might enable it to confer transgenerational memory epigenetic memory [44] (Figure 4).

Trans-acting small interfering RNAs (tasiRNAs) are a type of siRNA found in plants that are produced by being processed by miRNA. They are first transcribed from endogenous genes and have 5′ cap and 3′ polyA tail, which is later processed to form active tasiRNA by following cleavage by specific miRNAs. The cleaved fragment is converted into double-stranded RNAs by RNA-DEPENDENT RNA POLYMERASE 6 and then processed further by DCL4. TasiRNA then cleaves non-identical mRNA to play a role in gene silencing [45,46,47,48].

Plant miRNAs are usually 20–22 nucleotides long with a hairpin structure, transcribed from MIR genes by RNA polymerase II and later processed by DCL1, HYL1, and SE proteins to form a duplex RNA known as miRNA/miRNA* complex [49,50]. It is then methylated and transported to the cytoplasm where it is incorporated into AGO protein-containing RISC to find mRNA with a complementary sequence, leading to its destruction [51,52]. Though complementary mRNA degradation is the primary pathway for miRNA, in rice, slightly longer miRNA (24 nucleotides) is produced by DCL3 and associated with AGO4. It is found to cause transcriptional silencing of its own (cis) or other (trans) loci through cytosine methylation [53]. miRNA-mediated DNA methylation is also evident in Arabidopsis [54].

Long non-coding RNAs (lncRNAs) are longer than 200 nucleotides. They are transcribed by RNA polymerases II, IV, or V and after transcription, they undergo post-transcriptional modifications such as splicing and polyadenylation [55]. lncRNAs recruit chromatin-modifying complexes such as Polycomb Repressive Complex 2 (PRC2), which in turn methylates histone proteins (H3K27me3), which is a key step for silencing target genes [32]. lncRNAs can block transcription in one way by inhibiting the elongation step of RNA polymerase II; on the other hand, they bind with miRNAs to prevent them from silencing mRNA [55].

Besides ncRNAs, recent findings suggest that alternative splicing (AS) of stress-responsive genes also plays a role in stress memory response. For instance, under recurrent stress, certain alternatively spliced proteins reappear, indicating a form of post-transcriptional memory [56]. Also, plants were observed to accumulate substantial levels of intron retention (IR) events in certain genes during first exposure to stress, indicating repression of the splicing mechanism. These IR events were largely resolved during the subsequent recovery phase, suggesting a reactivation of normal splicing activity. However, upon a second heat stress exposure, the response of previously primed plants contrasted sharply with that of non-primed counterparts. While non-primed plants again exhibited high levels of IR, confirming continued splicing repression, primed plants maintained efficient splicing, closely resembling the splicing profiles seen under non-stressed conditions. This phenomenon, termed “splicing memory”, enables primed plants to recall prior stress exposure and adjust their splicing machinery accordingly, thereby promoting sustained growth and development even under repeated stress conditions [57].

## 3. Drought Stress Memory

In several plant species, expression of some miRNAs shows an increasing/decreasing pattern in subsequent stress, indicating memory response. They regulate the response of their target genes, which ultimately contributes to drought stress tolerance. For example, in wheat, 65 candidate drought memory-related miRNAs were experimentally validated through target gene cleavage analysis. These miRNAs influence key drought memory responses by regulating genes involved in signal transduction, transcription, and metabolic adaptation. Notably, miRNAs such as tae-miR531_L-2, when overexpressed in *Arabidopsis*, significantly enhanced drought tolerance, highlighting their functional relevance. miRNAs showing differential expression during recurring drought stress were also found to modulate the expression of genes involved in starch and sucrose catabolism, proline homeostasis, and reactive oxygen species balance—mechanisms critical for maintaining cellular function under recurring drought conditions [58]. In coffee plants too, upregulation of miRNA (such as miR408) has been observed in subsequent stress [59]. This miRNA is known to confer drought tolerance in *Medicago truncatuka* Gaertn. [60], *Hordeum vulgare* L. (barley) [61], and *Cicer arietinum* L. (chickpea) [62]. In rice *Oryza sativa* L., 238 lncRNAs showed memory-related expression in subsequent drought stress, including 12 potential miRNA precursors. For example, lncRNAs such as TCONS_00028567 show memory-responsive expression patterns and may give rise to miRNAs like osa-MIR1428e that regulate the hormone signaling pathway by regulating the SAPK10 protein gene [63].

**Figure 1:**
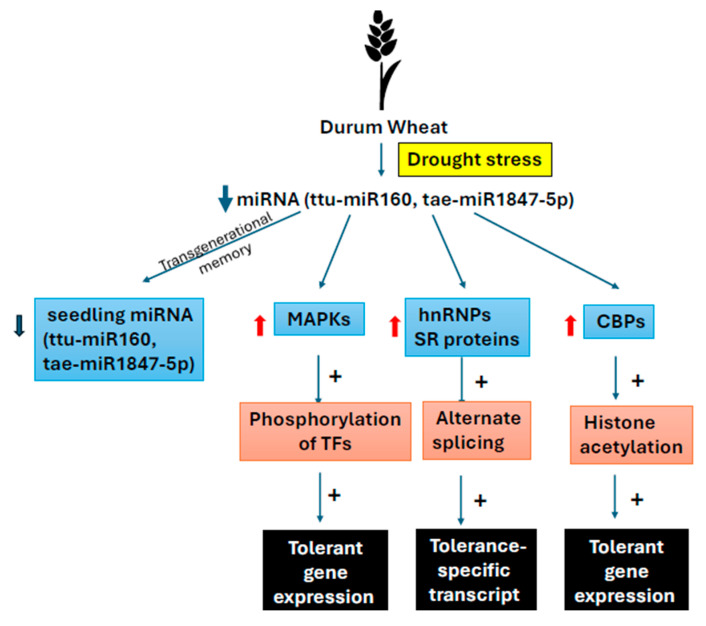
miRNA-mediated regulatory networks in drought stress memory of durum wheat.

Transgenerational drought memory in *Triticum turgidum* L. (durum wheat) has been associated with altered expression of miRNAs in progeny. Progenies of two types of drought stress-primed genotypes (drought-tolerant and drought-sensitive) show expression of various miRNAs. In the progeny of the tolerant genotype, more miRNA are upregulated than downregulated, while the opposite pattern is seen in the progenies of the sensitive genotype. The upregulated miRNA in the tolerant genotype probably inactivate genes playing a negative role in stress tolerance. Conversely, repressed miRNAs in sensitive genotypes leads to the de-repression of positive regulators, but potentially at the cost of slower or less targeted adaptation. Downregulated miRNAs in the tolerant genotype also play a role in transgenerational drought stress memory, possibly targeting three pathways. Firstly, the downregulation of specific miRNAs, such as *tae-miR1847-5p* in tolerant genotypes, led to the induction of CREB-binding proteins (CBPs), which modulate gene expression through histone acetylation and chromatin remodeling, aiding stress resilience. In contrast, its upregulation in sensitive genotypes likely impaired this epigenetic activation pathway [22]. Secondly, some downregulated miRNAs targeted mitogen-activated protein kinases (MAPKs), which play a crucial role in abiotic stress tolerance [64] by promoting signal transduction and root development via organizing cytoskeletal actin protein under drought stress [65]. Tolerant genotype-specific miRNAs (such as ttu-miR160, hvu-miR444b, and tae-miR1847-5p) were downregulated in the seedling, showing transgenerational drought stress memory, indicating uninhibited signal transduction by the MAPK signaling pathway. On the other hand, these miRNAs were increased or unchanged in the sensitive variety where the MAPK signaling pathway might have been repressed [22]. Thirdly, downregulation of specific miRNA promotes alternative splicing of some genes, which is a common scenario in plants’ stress tolerance response [66,67]. The serine/arginine-rich (SR) proteins and heterogeneous nuclear ribonucleoproteins (hnRNPs) are responsible for regulating the efficiency of splice site recognition [68]. Downregulation of ttu-miR160 in the stress-tolerant genotype is probably responsible for the upregulation of its target SR and hnRNP genes, potentially enhancing AS under transgenerational stress [22] (Figure 1).

## 4. Cold Stress Memory

Prolonged exposure to cold temperatures ensures that certain plants flower at the appropriate time by epigenetically silencing floral repressors. This process is known as vernalization. It is a form of epigenetic regulation that ensures flowering occurs at the appropriate time—typically after winter [69]. Cold-induced floral repressor during winter is mediated by *FLOWERING LOCUS C* (*FLC*) in *Arabidopsis*, which itself is repressed during spring (when the weather is warmer) to allow flowering [70]. The repression of *FLC* is achieved through a complex orchestration of epigenetic modifiers, transcription factors, and multiple cold-induced lncRNAs such as COOLAIR, COLDAIR, and COLDWRAP. Thus, flowering in spring is a manifestation of cold memory, where exposure to cold both repressed flowering through *FLC* and derepressed *FLC* through cold-induced lncRNAs.

**Figure 2:**
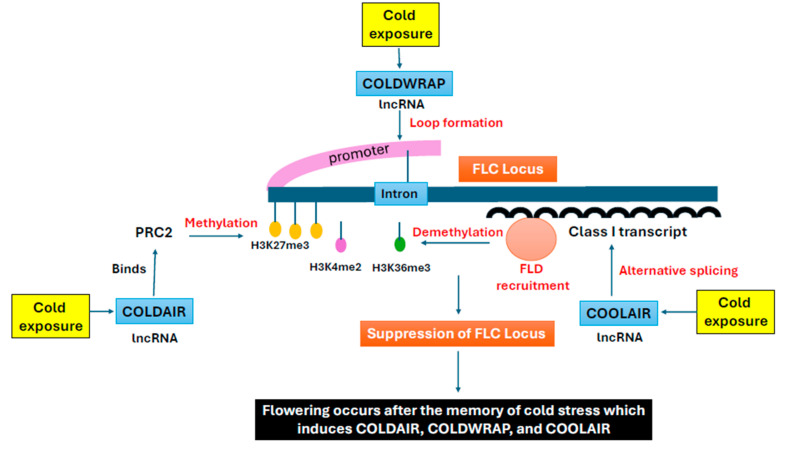
lncRNA-guided epigenetic silencing of *FLC* during vernalization.

*COOLAIR* is a cold-induced antisense transcript [71] which is physically associated with the *FLC* locus. COOLAIR promotes antisense-mediated transcriptional repression of the *FLC* locus, while PRC2 is responsible for epigenetic silencing [72]. Firstly, COOLAIR is alternatively spliced and polyadenylated, producing two major transcripts: class I with proximal polyadenylation and class II with distal polyadenylation. The prevalence of class I increases under warm temperatures which enhances H3K4me2 demethylase activity. This leads to demethylation of chromatin which condenses chromatin and reduces *FLC* transcription [73,74]. Secondly, besides the reduction in H3K36 methylation by COOLAIR, in spring, H3K27me3 is independently spread by Polycomb Repressive Complex 2 (PRC2) throughout the *FLC* locus. H3K27 methylation condenses chromatin, repressing the *FLC* locus. Thus *FLC*-mediated repression of flowering is removed in spring [75]. An increase in H3K27me3 and a parallel decrease in H3K36me3 is a hallmark of *FLC* repression [76]. PRC2 can spread H3K27me3 in the absence of COOLAIR but at a slower rate [72,75]. *FLC* mRNA is found to be very stable and balances the effects of short-term temperature fluctuations on *FLC* transcription, preventing premature flowering [75] (Figure 2).

Another lncRNA *COLDAIR* transcribed from the first intron of *FLC* after prolonged cold exposure (~20 days) binds directly to PRC2 components and guides them to the *FLC* nucleation site, thereby facilitating the deposition of H3K27me3 [77]. PRC2 components recruited by lncRNA act in concert with transcription factors like VAL1/VAL2 and chromatin proteins such as LIKE HETEROCHROMATIN PROTEIN 1 (LHP1) and bind to the cold memory cis-element (CME) of *FLC*. It causes methylation of H3K27 in the *FLC* locus, which promotes chromatin condensation and repression [78] (Figure 2).

*COLDWRAP*, another lncRNA, forms an intragenic chromatin loop connecting the *FLC* promoter with the 3′ end of its first intron, located downstream of the COLDAIR-transcribed region. This loop exists even before vernalization and becomes progressively stronger with prolonged cold exposure, suggesting it plays a role in maintaining stable repression of *FLC* during vernalization. This loop helps stabilize *FLC* repression by bringing the promoter and intronic regions together, reinforcing Polycomb silencing. In plants with reduced COLDWRAP expression, this loop formation is significantly weakened, highlighting COLDWRAP’s key role in facilitating this chromatin architecture [79] (Figure 2).

## 5. Heat Stress Memory

Induction of specific miRNA and alternative splicing is known to play roles in heat stress (HS) memory. Mutations in *ARGONAUTE1* (*AGO1*) and DICER-LIKE1 (DCL1) impair HS memory, confirming the role of miRNA in HS memory [80].

The most well-known miRNA playing a role in heat stress memory is miR156. It is among the most highly conserved miRNAs in the plant kingdom [81]. Though induction of this miRNA is observed after heat stress in mustard [82] and wheat [83], its role in heat stress memory is well documented in Arabidopsis. In Arabidopsis, miR156 isoforms (particularly miR156h) are strongly upregulated after heat stress, leading to post-transcriptional repression of their targets—SPL transcription factors (*SPL2*, *SPL9*, *SPL11*). Using a heat-inducible promoter, researchers confirmed that miR156 functions after the initial stress exposure, specifically during the memory phase, rather than during stress acquisition or basal thermotolerance. Overexpressing *miR156* enhances HS memory, while blocking it impairs recovery, demonstrating its necessity. Since *SPL* genes regulate developmental transitions, miR156-mediated repression delays flowering under stress, ensuring that resources are allocated to survival rather than reproduction. This mechanism suggests that miR156 acts as a molecular link between stress adaptation and developmental plasticity [80,84].

Another miRNA, miR824, and its target gene AGAMOUS-LIKE16 (*AGL16*) respond to recurring heat stress (HS) in plants, particularly in *Arabidopsis* and other Brassicaceae species. It was found that miR824 levels increase gradually under repeated mild heat stress (37 °C) through heat shock transcription factors HSFA1 and HSFA2. HSFA2 being one of the well-known heat stress memory genes [85] further confirms that miRNA plays an important role in heat stress memory. Mature miR824-loaded RISC cleaves AGL16, a MADS-box transcription factor maintaining its repression post stress. AGL16 represses *FLOWERING LOCUS T* (*FT*), a key floral integrator. Heat-induced miRNA-mediated downregulation of *AGL16* mildly derepresses *FT*, potentially fine-tuning flowering time [86].

**Figure 3:**
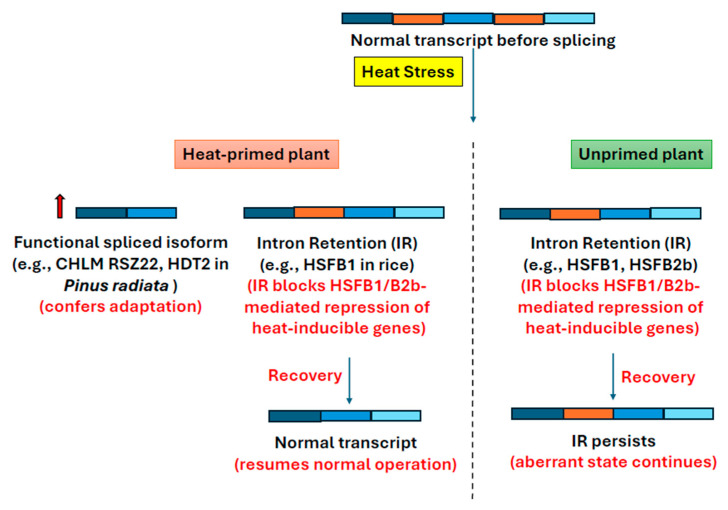
Splicing memory dynamics in heat stress responses. The red arrow indicates increased amount of isoform.

Alternative splicing (AS) serves as a critical regulatory mechanism under heat stress, where intron retention (IR) occurs to prevent the production of aberrant proteins or peptides, thereby reducing the burden on the proteasome machinery. In the primed Arabidopsis plant, the AS pattern resets to baseline level after recovery, whereas non-primed plants retain IR isoforms, leading to impaired growth. During subsequent heat stress, primed plants maintain efficient splicing, while non-primed plants accumulate IR transcripts, highlighting a splicing-based memory mechanism (Figure 3). Notably, class B heat shock factors (HSFB1, HSFB2b) exhibit higher IR during both priming stress and recurrent stress (in the primed plants), whereas heat-induced class A HSFs (HSFA2, HSFA7a) remain less affected, promoting thermotolerance [57]. Class B HSFs are known to repress heat-inducible genes under non-stressed conditions and during the post-stress recovery period [87]. Thus, inactivation of class B HSFs is crucial to achieve heat stress recovery. This splicing memory enables primed plants to rapidly restore normal gene expression post stress, while non-primed plants remain in a dysfunctional, stress-like state. Furthermore, over 65% of genes with differential intron retention (DIR) also show differential expression in Arabidopsis, indicating coordination between AS and transcriptional regulation. DIR genes are enriched in pathways such as protein folding, RNA processing, and abiotic stress responses, underscoring the role of splicing in stress adaptation [57].

*Pinus radiata* exhibit long-term splicing memory, a conserved yet mechanistically distinct response to heat stress independent of intron retention (IR). Several proteins such as *CHLM* (responsible for chloroplast–nucleus coordination), *RSZ22* (*responsible for* RNA processing), and *HDT2* (*responsible for* epigenetic modulation) are alternatively spliced during initial heat exposure, which may persist for at least six months. These findings suggest that splicing memory buffers metabolic costs by maintaining stress-adapted transcript isoforms, potentially enhancing translational efficiency during recurrent stress. Notably, this memory operates independently of intron retention (IR). Instead, *P. radiata* favors exon skipping and other splicing modalities, likely due to its long introns, which may impede full-length IR and promote fragmented retention [56] (Figure 3).

Heat stress can often activate silenced transposons such as a copia-like retrotransposon named ONSEN in Arabidopsis. ONSEN contains heat stress response elements in its long terminal repeat, and thus, it is activated during heat stress probably by the action of the HSFA1 and HSFA2 transcription factors. Though ONSEN hijacks the plant heat stress response, it has other advantages. Mutants defective in siRNA biogenesis (nrpd1 mutant) exhibited transgenerational transposition when exposed to heat stress (HS) during early vegetative growth [88] (Figure 4). The retrotransposition (amplified DNA copies of ONSEN) occurs in flowers before gametogenesis, causing genetic variability through insertional mutation, increasing the possibility of producing heat-resistant offspring even in progenies derived from tissues that differentiated after HS exposure [84,88,89]. These findings suggest that transpositional activity can be stably maintained in undifferentiated cells long after the initial HS trigger. Retrotransposition was not observed in the progeny of heat-primed plants with intact siRNA biogenesis pathways or in non-primed siRNA mutants [90]. It indicated the crucial role of the siRNA pathway in restricting stress-induced retrotransposition.

In Arabidopsis, besides miRNA and AS, tasiRNA also plays a role in heat stress memory. The biogenesis of tasiRNAs critically depends on the gene *SUPPRESSOR OF GENE SILENCING 3* (*SGS3*); degradation of SGS3 leads to a marked reduction in tasiRNA levels. Under normal conditions, tasiRNAs suppress the expression of target genes such as *HEAT-INDUCED TAS1 TARGET 5* (*HTT5*), which modulates both flowering and immune responses. During heat stress, the transcription factor HSFA2 induces the expression of the E3 ubiquitin ligase SGIP1, an enzyme typically involved in ubiquitin-mediated degradation of SGS3. When SGS3 is reduced, the biogenesis of tasiRNA is hampered. Thus, heat-induced tasiRNA suppression derepresses *HTT5*, promoting early flowering at the cost of attenuated disease resistance. Strikingly, this epigenetic reprogramming persists in unstressed progeny, demonstrating transgenerational memory of heat adaptation. Early flowering ensures reproductive success under recurrent heat stress, while attenuated immunity may reflect a trade-off favoring rapid lifecycle completion [91].

## 6. Heavy Metal Stress Memory

Studies by Cong et al., Rahavi et al., and Ou et al. collectively propose a model in which small interfering RNAs (siRNAs) may play a role in mediating the transgenerational memory of heavy metal stress in rice and Arabidopsis. In these studies, exposure to heavy metals (such as Cd, Cr, Hg, Ni, and Cu) caused the CHG hypomethylation of genes related to heavy metal pathways and TEs (such as Tos17), higher homologous recombination frequency (HRF), higher expression of heavy metal transporter genes (OsHMAs), and these patterns were carried across multiple generations [6,18]. The progeny generation also showed higher resistance to heavy metal stress, such as longer plant height under stress, longer root length, and higher chlorophyll content and even cross-tolerance to salt (NaCl) and methyl methane sulfonate (MMS), indicating transgenerational memory response [18,92]. Ou et al. observed downregulation of one isoform of CMT3 in progeny generation, the primary role of which is to reinforce CHG methylation after DNA replication before cell division. In the progeny, CHG hypomethylation was associated with reduced expression of *CMT3*—a key maintenance methyltransferase—and increased expression of demethylases such as DNA glycosylase *DME* [92] (Figure 4). It is known that siRNA causes methylation in DNA in a pathway known as RNA-dependent DNA methylation (RdDM). Rahavi et al. suggested that differential siRNA expression under heavy metal stress was responsible for the alteration of expression of components of the RdDM pathway, which ultimately resulted in the hypomethylation of heavy metal transporter genes (OsHMAs) and TEs. Thus, removal of suppression of gene expression by hypomethylation resulted in higher expression of heavy metal transporters, which helped plants by sequestering toxic ions into vacuoles (OsHMA4) or effluxing excess metals (OsHMA9), thereby reducing cellular toxicity in rice [6]. Though hypomethylation was observed in the TEs, there is not enough evidence of whether hypomethylation was sufficient to activate them to undergo transposition, a phenomenon observed in heat stress [6,18,92]. In heat stress, the transposon becomes active and creates multiple copies in progenies This activation may represent a stress-induced increase in genetic variability via insertional mutagenesis, potentially contributing to stress resilience in future generations.

## 7. Salt Stress Memory

RNA-directed DNA methylation (RdDM) and lncRNAs have been found to play roles in mediating transgenerational salt-induced osmotic stress in *Arabidopsis thaliana*.

Wibowo et al. (2016) demonstrated that repeated exposure to hyperosmotic stress caused hyper- (CHG and CHH methylation context) and hypo-methylation of DNA in *Arabidopsis thaliana*, including many epigenetically labile sites and transposable elements (TEs), which were inherited epigenetically in progeny through maternal germlines and improved germination and survival rates. The differentially methylated regions (DMRs) contain many genes responsive to salt stress. The DMRs could be responsible for the differential expression of these genes that contributed to the better response to salt stress in progeny. The role of DMR is further proved by the fact that mutants lacking the components of RdDM pathways (such as cmt, drm, nrpd, and dml mutants) failed to transfer the transgenerational salt stress memory to the progenies [93] (Figure 4). For example, a key gene involved in hyperosmotic stress tolerance is CNI1, which is regulated by a stress-induced antisense lncRNA called CNI1-AS1 and a downstream regulatory region termed the hyperosmotic stress-induced differentially methylated region (HS-DMR). CNI1-AS1 is transcribed in the opposite direction of CNI1 under stress and contributes to the repression of CNI1 expression. Hypomethylation of this HS-DMR decreased the expression of CNI1-AS1, thus increasing CNI1 expression. CNI1 (*Carbon/Nitrogen Insensitive 1*) is a plant-specific RING-type E3 ubiquitin ligase that plays an important role in carbon/nitrogen (C/N) nutrient balance, drought tolerance, and pathogen defense by tagging specific regulatory proteins for ubiquitination and degradation. It thus modulates critical signaling pathways that control growth, senescence, and immune responses under environmental stress conditions [94,95,96,97,98,99,100].

Another interesting fact is that this transgenerational salt stress memory is strictly transferred via the maternal germline since mother and progeny plants are more likely to live in the same stressful environment. On the other hand, DNA demethylation enzymes actively erase stress-induced marks in the male germline (pollen), limiting paternal inheritance. However, in dml mutants, demethylation is impaired, allowing paternal transmission of stress memory, confirming the importance of germline-specific epigenetic erasure [93].

## 8. Biotic Stress Memory

Transgenerational memory of biotic stress tolerance has been observed in various studies. For example, infection with avirulent *Pseudomonas syringae* and treatment with β-aminobutyric acid primed Arabidopsis for the induction of SA-mediated defense responses in the subsequent generation [101]. In another study, the descendants of primed plants showed a faster and stronger SA-mediated defense response relative to controls [102].

Small interfering RNAs (siRNAs) play a critical role in the transgenerational memory of insect resistance in plants. In Arabidopsis and tomato, parent plants primed with caterpillar herbivory, methyl jasmonate treatment, or mechanical damage conferred transgenerational insect resistance on progeny. This inherited resistance is absent in Arabidopsis mutants deficient in siRNA biogenesis—specifically, *dcl* and *nrpd*—indicating that functional siRNA pathways are essential for transmitting this defense memory [23]. siRNAs—processed by DICER-LIKE (DCL) enzymes and dependent on Nuclear RNA Polymerase D (nrpd/Pol V)—are mobile via the phloem, transmitting stress signals from damaged tissues to developing seeds. Within progeny, siRNAs may guide epigenetic modifications, such as DNA methylation or mRNA degradation, thereby priming defense genes for rapid activation upon insect attack. The fact that resistance is inherited through the embryo rather than maternal tissues supports the model of siRNA-mediated reprogramming of the germline. These findings suggest that siRNAs act as mobile, sequence-specific messengers of environmental experience, enabling plants to encode and transmit adaptive insect resistance across generations [23].

## 9. Conclusions

This review summarizes the role of non-coding RNAs in memory responses to drought, salt, heavy metal, biotic, heat, and cold stress. The findings highlight how they enable plants to establish, maintain, and transmit stress memory, enhancing their adaptability in an environment susceptible to recurrent stress. It is important to note that these non-coding RNAs are expressed in a time- and space-dependent manner and can be mobile, allowing them to coordinate systemic stress responses [103,104]. Though most studies have focused on the role of only one type of non-coding RNA in a particular type of stress, it is possible that they act synergistically to fine-tune stress responses. For example, miRNAs and siRNAs have been found to play a role in heat stress memory, though in separate studies [80,91]. Thus, the activation of one RNA class does not exclude the possible involvement of another class.

Understanding these regulatory mechanisms may reveal novel targets for engineering stress-tolerant crops. For example, the genes that give rise to non-coding RNAs conferring stress memory can be upregulated through genetic engineering to obtain plants with enhanced memory responses. Knowledge of potential non-coding RNAs can also be implemented during breeding to obtain stress-tolerant plant varieties. Since these non-coding RNAs can also be used as potential biomarkers, their higher expression can be used to readily identify plants with stress memory.
